# Pathogenicity and Antibiotic Resistance Diversity in *Clostridium perfringens* Isolates from Poultry Affected by Necrotic Enteritis in Canada

**DOI:** 10.3390/pathogens12070905

**Published:** 2023-07-03

**Authors:** Sara García-Vela, Agustí Martínez-Sancho, Laila Ben Said, Carmen Torres, Ismail Fliss

**Affiliations:** 1 Department of Food Science, University of Laval, Quebec, QC QCG1V0A6, Canada; sara.garcia-vela1@ulaval.ca (S.G.-V.); laila.ben-said1@ulaval.ca (L.B.S.); 2 Area of Biochemistry and Molecular Biology, OneHealth-UR Research Group, University of La Rioja, 26006 Logrono, La Rioja, Spain; agusti.martinez@unirioja.es

**Keywords:** necrotic enteritis, *Clostridium perfringens*, antimicrobial resistance, *erm*(T), bacteriocin genes, *bcn5*

## Abstract

Necrotic enteritis (NE) caused by *C. perfringens* is one of the most common diseases of poultry and results in a huge economic loss to the poultry industry, with resistant clostridial strains being a serious concern and making the treatment difficult. Whole-genome sequencing approaches represent a good tool to determine resistance profiles and also shed light for a better understanding of the pathogen. The aim of this study was to characterize, at the genomic level, a collection of 20 *C. perfringens* isolates from poultry affected by NE, giving special emphasis to resistance mechanisms and production of bacteriocins. Antimicrobial resistance genes were found, with the *tet* genes (associated with tetracycline resistance) being the most prevalent. Interestingly, two isolates carried the *erm(T)* gene associated with erythromycin resistance, which has only been reported in other Gram-positive bacteria. Twelve of the isolates were toxinotyped as type A and seven as type G. Other virulence factors encoding hyaluronases and sialidases were frequently detected, as well as different plasmids. Sequence types (ST) revealed a high variability of the isolates, finding new allelic combinations. Among the isolates, *C. perfringens* MLG7307 showed unique characteristics; it presented a toxin combination that made it impossible to toxinotype, and, despite being identified as *C. perfringens*, it lacked the housekeeping gene *colA*. Genes encoding bacteriocin BCN5 were found in five isolates even though no antimicrobial activity could be detected in those isolates. The *bcn5* gene of three of our isolates was similar to one previously reported, showing two polymorphisms. Concluding, this study provides insights into the genomic characteristics of *C. perfringens* and a better understanding of this avian pathogen.

## 1. Introduction

Necrotic enteritis (NE) caused by *Clostridium perfringens* is one of the most common diseases of poultry and results in a huge economic loss to the poultry industry [1]. A distinguishing feature of NE is acute death, with mortality rates as high as 50%. Clinical symptoms include depression, dehydration, drowsiness, ruffled feathers, diarrhea, and reduced feed consumption [2]. The subclinical form of the disease causes chronic damage to the intestinal mucosa in chickens, resulting in poor absorption of nutrients, reduced weight gain, and a decrease in overall performance. In healthy chickens, *Clostridium perfringens* can be found at low levels in the intestines (<10^5^ CFU/g), but this level may increase, and poultry become prone to NE [1].

*C. perfringens* is a Gram-positive, spore-forming, strictly anaerobic bacterium that can be found in a variety of environments, including food, soil, and in the gastrointestinal tracts of both diseased and healthy animals and humans [3]. It is a widespread pathogen that can be classified as toxin types A–G, depending on the combination of the following toxins: α-toxin, β-toxin, ε-toxin, ι-toxin, enterotoxin (CPE), and NetB. This microorganism also produces other toxins which are not considered for typing. These include β2-toxin, λ-toxin, and θ-toxin [4,5]. Hence, they produce a diversity of diseases in both animal and human hosts [3,4,5].

Toxinotype G is a proven cause of NE in chickens [6], in which NetB plays an important role. It is a plasmid-encoded, pore-forming toxin exclusive for *C. perfringens* coming from poultry affected by NE. It is a key virulence factor in the pathogenesis and is similar to *S. aureus* alpha-hemolysin. It forms heptameric pores on its target cell membranes [7,8]. Sequences of *netB* genes from isolates from around the world show that the coding sequence is highly conserved across all strains [9]. Other toxins present in *C. perfringens* from poultry with NE are α-toxin, β2-toxin, and θ-toxin. The α-toxin is a secreted zinc-metalloenzyme with lethal, hemolytic, and dermonecrotic activities, as well as phospholipase C and sphingomyelinase activities, and it is a major pathogenic factor in the development of gas gangrene. At low doses, it causes limited phospholipid hydrolysis, which in turn activates diacylglycerol- and ceramide-mediated signaling pathways, leading to cell apoptosis [10,11,12]. The β2-toxin has no significant homology with the sequence of β-toxin or any other known protein sequence, and its mechanism is still unknown [13,14]. The θ-toxin is a cholesterol-dependent cytolysin and is a member of the β pore-forming family of toxins [15].

*C. perfringens* also carries other virulence genes such as those encoding sialidases, exoenzymes, and adhesion proteins. The most common degradative enzymes are proteases (e.g., clostripain), hyaluronidase (mu-toxin), collagenase, endoglycosidases, and the sialidases NanJ, NanI, and NanH (neuraminidases), which generate free sialic acids [16].

Antimicrobial resistance is also a concern in infections caused by *C. perfringens*. The continued widespread use of antibiotics in poultry during the last years has led to changes in the bacterial environment, eliminating susceptible strains and allowing antimicrobial-resistant bacteria to persist and predominate. Antibiotics have been used as growth promoters for decades, although this practice is now banned in many countries [17]. Antimicrobial resistance, together with a gradual decrease in the susceptibility of some strains of *Eimeria* spp. to anticoccidial agents (a predisposing factor for NE), can lead to an increase in the occurrence of *C. perfringens* strains [18]. Acquired antimicrobial resistance genes are commonly plasmid-associated. Plasmid-carrying tetracycline resistance genes (*tet*) are frequent [19], as well as those related to macrolide and lincosamide resistance (mainly erythromycin and lincomycin) [3]. Multidrug resistance among *C. perfringens* isolates has been described in different studies. Resistance to tetracycline, lincomycin, enrofloxacin, cefoxitin/ampicillin, and erythromycin via the detection of *tet*, *Inu*, *qnr*, *bla*, and *erm*(B) genes, respectively, has been identified in *C. perfringens* of foodborne infections by PCR in Egypt [20]. This phenomenon is also frequent in *C. perfringens* coming from birds, as well as in those coming from other sources. However, many studies only include the phenotypic detection of antimicrobial resistance [21,22]. Thus, further studies are needed to determine the current status of resistance genetic profile in *C. perfringens*.

The whole-genome sequencing (WGS) approach could be a good tool for this purpose. In this respect, the *C. perfringens* genomes of isolates of different locations and sources (including strains from cattle, dogs, and horses) were previously analyzed by WGS to assess their genetic diversity and phylogenetic relatedness [23]; this study established that the genetic diversity of *C. perfringens* is based on a large number of virulence factors that vary among phylogroups and antibiotic resistance markers. These methods may help to develop future strategies to prevent disease caused by this emerging and poorly understood pathogen.

The production of antimicrobial peptides, such as bacteriocins, by *C. perfringens* has also been reported. This trait is sometimes considered virulence factors, as they could inhibit the growth of not only pathogenic bacteria, but also commensals for competition with the ecological niche in the host gut. Bacteriocin BCN5 and perforin are the well-known plasmid-encoded bacteriocins produced by *C. perfringens* [4,16]. Recently, the structural gene of Lactococcin A has been detected in a *C. perfringens* strain from poultry [24]. More in-depth studies are necessary for the study of bacteriocin production in *C. perfringens* and the possible link with the virulence of this pathogen.

The aim of this study was to characterize via WGS a collection of *C. perfringens* isolates of poultry affected by NE, giving special emphasis to the characterization of antimicrobial resistance determinants, as well as to the presence of virulence and bacteriocin genes and its correlation with the expression of antimicrobial activities by the isolates. 

## 2. Materials and Methods

### 2.1. Strain Collection, Maintenance and Propagation

A collection of 20 *C. perfringens* isolates, previously recovered from poultry affected by NE, and belonging to the University of Laval collection (Quebec, QC, Canada), was included in this study. *C. perfringens* ATCC 13124 was used as a control strain. The isolates were preserved in glycerol 40% at −80 °C. A reinforced medium for clostridia (HiMedia, Kelton, PA, USA) was used for the propagation of the isolates (incubation at 37 °C, 24 h, under strict anaerobic conditions).

### 2.2. Antibiotic Susceptibility Testing

Antibiotic susceptibility testing was performed by calculating the minimal inhibitory concentration (MIC) with the 20 *C. perfringens* isolates following the recommendations of the Clinical and Laboratory Standard Institute [25]. The following antibiotics were tested: ampicillin, cefotaxime, imipenem, tetracycline, chloramphenicol, clindamycin, metronidazole, and erythromycin. The strains were then identified as susceptible (S), resistant (R), or intermediate (I) in accordance with the protocol interpretation guidelines [25].

### 2.3. Screening for Bacteriocinetic Activity

The antimicrobial activity of the collection of *Cloatridium perfringens* isolates was studied using the *spot-on the lawn* method and agar well diffusion, as previously described [26]. In the case of *spot-on the lawn* method, the following indicator bacteria were used: *Clostridium tyrobutyricum* ATCC25755, *Pediococcus acidilactici* UL5, and *Enterococcus faecalis* ATCC29212. The same indicator bacteria were used for the well diffusion method, but *Micrococcus luteus* ATCC10240 was also included. For the *spot-on the lawn* method, tryptic soy agar plates were used with a thin layer of tryptic soy broth supplemented with 8% agar and 3% yeast extract that was inoculated with the indicator bacteria. The medium used for agar well diffusion was “reinforced medium for clostridium” supplemented with 8% agar for the indicator strain *Clostridium tyrobutyricum* ATCC25755, Tryptic Soy Agar for *Enterococcus faecalis* ATCC29212, MRS supplemented 8% agar for *Pediococcus acidilactici* UL5, and Nutrient Broth supplemented 8% agar for *Micrococcus luteus* ATCC10240.

### 2.4. Whole-genome Sequencing (WGS) Analysis

DNA was extracted using the DNeasy Blood and Tissue Kit (QIAGEN, Hilden, Germany) according to the manufacturer’s instructions for Gram-positive bacteria. The DNA was subjected to WGS using the Illumina technique at the Hospital Center of the University of Laval (CHUL), Quebec, Canada.

Raw sequencing data were processed using fastp 0.20.0 for trimming and quality control of trimmed reads [27]. De novo assembly, without alignment to a reference genome, was performed with SPAdes 5.0.2 [28], using QUAST 1.14.6 for checking the assembled quality [29]. Prokka 1.14.6 [30], which uses Prodigal for prediction of coding sequences [31], was used for gene prediction and annotation.

For detection of genes associated with antibiotic resistance, ResFinder 4.1 was used [32,33,34]. For plasmid detection, the program PlasmidID 1.6.4 [35] was used. Genes encoding virulence factors were detected using the ABRicate 1.0.1 program with the VFDB database [36]. Toxinoytpe assignment was performed using TOXIper v1.1 [37].

Multi-locus sequence typing (MLST) was tested in the genome data using MLST 2.0 [38,39,40,41,42,43]. The representation of phylogenetic relationships in a tree was performed using R version 4.2.1 [44], and phylogenetic distances were calculated using the average nucleotide identity (ANI) method, calculated with pyANI, a program that uses the ANI for whole-genome comparisons, and renders graphical summary output [45]. The graphic was generated using R version 4.2.1 [44] with ape 5.0 [46].

For the detection of bacteriocin genes, antiSMASH 7 beta [47] and BAGEL4 [48] were used. Blastp of the secondary metabolites detected was performed for identification of the peptides produced. Jalview 2.11.2.5. [49] and Clinker [50] were used to align bacteriocin genes detected among the *C. perfringens* species and to compare their genetic environments, respectively. GenBank database was used to obtain genes and plasmids of reference.

Multiple sequence alignment and visualization of the *erm*(T) gene products and the *bcn5* products, as well as generation of phylogenetic relationships between the *erm*(T) and *bcn5* products of our *C. perfringens* isolates and those of other bacterial species was established with the program Jalview 2.11.2.5 [49]. The representation of the genetic environment of *erm*(T) and *bcn5* genes in comparison with other genetic environments of *erm*(T) present in different bacterial species and a reference plasmid carrying the *bcn5* gene, respectively, was performed using the program Clinker [50]. The *erm*(T) genes from other bacterial species and the *bcn5* genes and genetic environments from other *C. perfringens* isolates were obtained from the GenBank databases.

## 3. Results

### 3.1. Resistance Phenotype

The rates of antibiotic resistance in the collection of 20 *C. perfringens* isolates were as follows (Table 1): tetracycline (50%; MIC ≥ 16 µg/mL), clindamycin (40%; MIC ≥ 8 µg/mL), and cefotaxime (5%; MIC ≥ 64 µg/mL); no resistant isolates were detected for metronidazole, chloramphenicol, ampicillin, and imipenem. Nevertheless, isolates in the intermediate susceptibility category were identified for tetracycline (15%; MIC 8 µg/mL), clindamycin (25%; MIC 4 µg/mL), and ampicillin (5%, MIC 1 µg/mL). In the case of erythromycin, there are no breakpoints in CLSI to classify isolates as resistant, intermediate, or susceptible for this agent. However, most of the isolates showed a MIC for ERY of 1–16 µg/mL, and only three isolates showed a very high MIC value (>128 μg/mL). According to these data, we consider these last three isolates as ERY-resistant (15%).

### 3.2. Whole Genome Sequencing

#### 3.2.1. Resistome

Antimicrobial resistance genes (ARG) were found in 16 of the 20 isolates analyzed (Table 2). Those isolates in which no resistance genes were detected were *C. perfringens* MLG2203, susceptible to all antibiotics tested, *C. perfringens* MLG1819 and MLG1819, resistant to clindamycin, and *C. perfringens* MLG7307, which was resistant to clindamycin and cefotaxime and presented intermediate susceptibility to ampicillin. Different tetracycline resistance genes (*tetA*, *tetB*, and *tet44*) were found in 16 of the isolates. The *InuP* gene was found in three isolates from our collection, two of them resistant to clindamycin.

The *erm*(T) gene was detected in two of our strains (*C. perfringens* MLG1108 and MLG 7009), and both strains showed very high MIC values for erythromycin (>128 μg/mL). Nevertheless, another additional *C. perfringens* isolate (MLG3111) showed resistance to this antimicrobial agent (MIC > 128 μg/mL), but it lacked known erythromycin resistance genes (Table 1 and Table 2).

**Figure 1:**
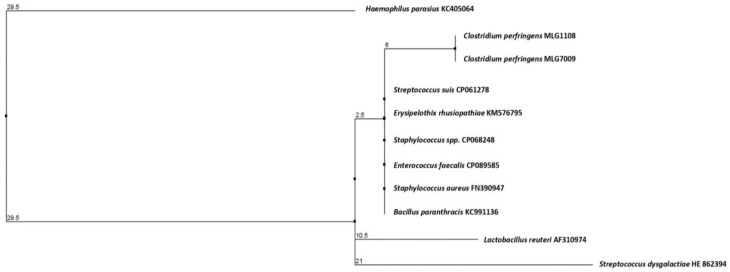
Phylogenetic relationships of the *erm*(T) gene present in *C. perfringens* MLG1108 and MLG7009 strains and in those of other different Gram-positive species. Sequences of the *erm*(T) gene from different strains were obtained from GenBank database and were grouped in a tree according to their average phylogenetic distances with the program Jalview 2.11.2.5. Numbers indicate phylogenetic distances.

Figure 1 shows phylogenetic relationships and alignments of the *erm*(T) product (metyltransferase) found in *Clostridium perfringens* MLG1108 and MLG7009 (which were identical) with the metyltransferases from other Gram-positive bacteria. When comparing the methylases encoded by the *erm*(T) gene of our *Clostridium perfringens* strains with those of others strains such *as Streptococcus suis* CP061278, *Erysipelothix rhusiopathiae* KM576795, *Staphylococcus* spp. CP068248, *Staphylococcus aureus* FN390947, *Enterococcus faecalis* CP089585, and *Bacillus paranthracis* KC991136, we spotted only one difference in the amino-acid sequences. Indeed, the lysine at position 30 of the *Clostridium perfringens* methylase was replaced by a threonine (Lys30Thr) in the ErmT sequence of the other Gram-positive bacteria. A comparison with the ErmT sequence of *Lactobacillus reuteri* AF310974 revealed three substitutions: Lys30Thr, Arg204Ile, and Leu476Phe. With respect to the *Streptococcus dysgalactiae* HE862394 methylase, we detected the Lys30Thr substitution plus two deletions present at positions 74 and 75 in the *Streptococcus dysgalactiae* methylase. Lastly, 16 amino-acid substitutions were identified in the *Haemophilus parasius* KC405064 methylase respect to that of *Clostridium perfringens* (Supplementary Figure S1).

**Figure 2:**
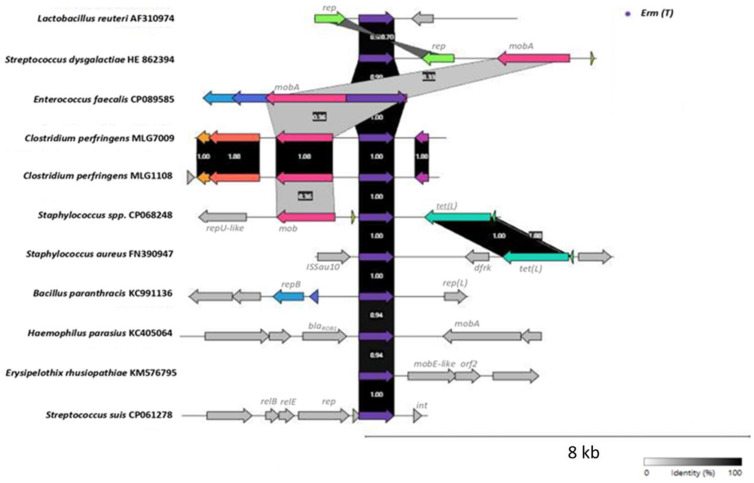
Comparison of genetic environments on *erm*(T) gene from *C. perfringens* strains MLG1108 and MLG7009 with the genetic environments of *erm*(T) from other Gram-positive bacteria. Different genes are indicated with arrows. Colors in the arrows represent the genes which shows similarities, and identities between them are indicated with numbers and a scale of gray. The *erm*(T) gene from different bacteria is represented with the purple arrow.

Figure 2 shows a comparison of the genetic environments of the *erm*(T) gene from other Gram-positive bacteria with those of our *C. perfringens* isolates. As we can see, the genetic environments of our *C. perfringens* isolates MLG1108 and MLG7009 were identical to each other and had few similarities with other genetic environments previously described. Only *mob* and *moba* genes were found to be 36% identical to those in *Staphylococcus spp.* CP068248 and *E. faecalis* CP089585. The remainder of the predicted genes in the genetic environment on the *C. perfringens erm*(T) gene showed no similarities with those contemplated for other genetic environments. Overall, we can see that the *erm*(T) gene is highly preserved among different species, but its genetic environments are very different from one species to another.

The aminoglycoside resistance gene *ant(6)-Ib* was found in *C. perfringens* MLG2314, being the first report in which *ant(6)-Ib* gene is reported in a toxitype A *C. perfringens* isolate.

Interestingly, no resistance genes were detected by WGS in *C. perfringens* MLG7307.

#### 3.2.2. Toxinotyping, Virulence Factors, and Plasmidome

**Figure 3:**
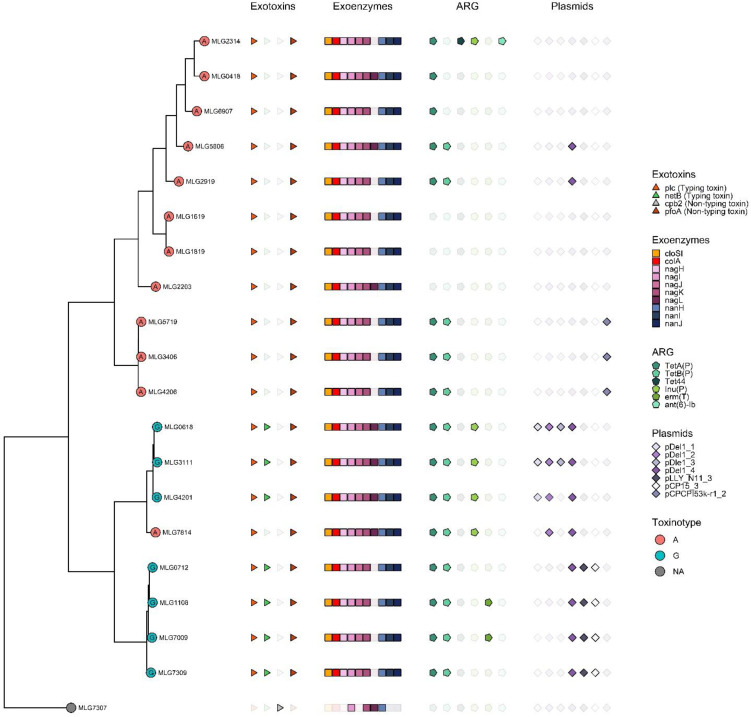
Phylogenetic relationships and main features of the 20 *C. perfringens* isolates; toxinotypes, exotoxins produced, exoenzymes, antimicrobial resistance genes (ARG), and plasmids detected. Letters A and G indicate toxinotype A and G, respectively. Different shapes of the symbols colored or uncolored indicate different genes detected. Triangles indicate exotoxins used for toxinotyping, Squares indicate presence of genes encoding exoenzymes: in yellow, *cloSI*, encoding the alpha-clostripain; in red, *colA* encoding the kappa-toxin; in purple, *nagH*, *nagI*, *nagJ*, *nagK*, and *nagL*, encoding the mu-toxin; in blue, *nanH, nanI*, and *nanK*, encoding the sialidases. Pentagons correspond to ARG, and purple diamonds indicate the presence of different plasmids.

Twelve of the *C. perfringens* isolates were toxinotyped as type A, carrying the *plc* gene encoding α-toxin. Seven of the strains were toxinotyped as type G, carrying *netB* in addition to *plc*. The gene of the non-typing toxin PFO (*pfoA*) was detected in both A and G toxinotypes. Figure 3 shows the phylogenetic relationships, toxinotyping, plasmids, antimicrobial resistance genes (ARG), and main virulence factors detected in our collection of *C. perfringens* isolates. *C. perfringens* MLG7307 could not be toxinotyped since it did not carry the *plc* gene, present in all toxinotypes. Instead, it carried the genetic determinants for the non-typing β2-toxin (*cpb2*). Other virulence factors such as *cloSI* and *colA* were present in the majority of the isolates, except for *C. perfringens* MLG7307. Other virulence factors detected in our collection include the genes of mu-toxin and of the three sialidases NanH, NanI, and NanJ.

Thirteen of the isolates carried at least one plasmid. Plasmids detected are represented in Figure 3.

Table 3 includes the sequence type (ST) of the isolates, new allelic combinations, and alleles with <100% identity or coverage of the isolates. A sequence type could be established for nine of the isolates, with ST73 (*n* = 4) and ST21 (*n* = 4) being the most prevalent, followed by ST279 (*n* = 1). Three additional isolates showed two new allelic combinations. Moreover, another six isolates showed alleles with <100% identity, suggesting the existence of new alleles and, as a consequence, of new STs. Another isolate showed an allele (*sigk*) with <100% of coverage, and ST could not be assigned. Lastly, *C. perfringens* MG7307 could not be typed because it lacked the housekeeping *colA* gene.

#### 3.2.3. Secondary Metabolites

Genes encoding secondary metabolites were detected in all isolates (Table 4). Among them, genes encoding bacteriocin BCN5, sactipeptides, lassopeptides, RiPP-like (ribosomally synthesized and post-translationally modified peptides), and NRS-like (non-ribosomal peptide synthesized) were identified. A bacteriocin-like peptide was also detected in one isolate, *C. perfringens* MLG4206.

**Figure 4:**
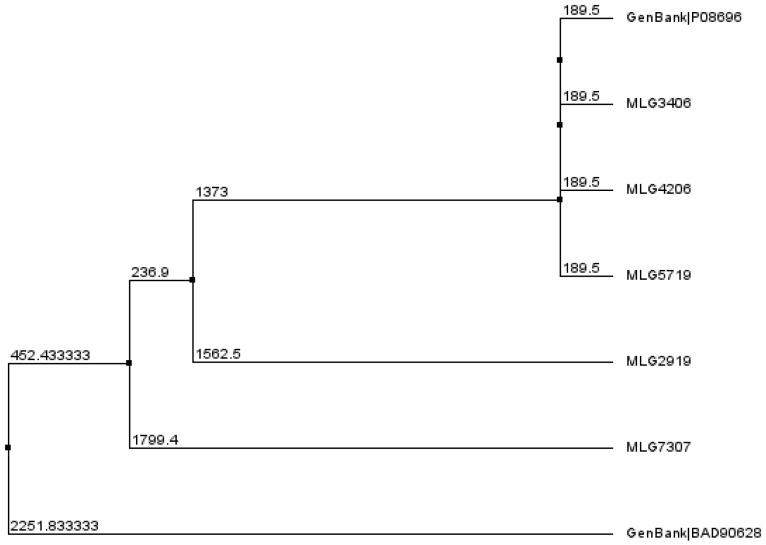
Alignments of the bacteriocin BCN5 from the five isolates of our collection (*C. perfringens* MLG3406, MLG4206, MLG5719, MLG2919, and MLG7307) and two bacteriocin BCN5 from GenBank database (P08696 and BAD90628). Numbers indicate phylogenetic distances. Tree generated with Jalview 2.11.2.5.

The bacteriocin BCN5 was detected in five of our isolates. Figure 4 represents the phylogenetic relationships and distances among the bacteriocin BCN5 present in the five *C. perfringens* isolates and two bacteriocin BC5 of reference. Alignments of the BCN5 at the amino-acid level can be found in Supplementary Figure S2. Bacteriocin BCN5 from MLG3406, MLG4206, and MLG5719 presented a length of 890 amino acids; they were identical among themselves and to bacteriocin BCN5 P08696 from the GenBank database. They showed a 65.2% of identity with bacteriocin BCN5 from MLG2919, which presents a length of 910 amino acids. Comparing the BCN P08696 with the bacteriocin BCN5 of *C. perfringens* MLG7307, whose length is 577 amino acids, the identity was 93.89%. The other bacteriocin of reference considered from GenBank databases (accession number BAD90628) was phylogenetically closer to BCN5 from *C. perfringens* MLG7307 and MLG2919, and it presented just 50.3% identity with BCN P08696.

**Figure 5:**
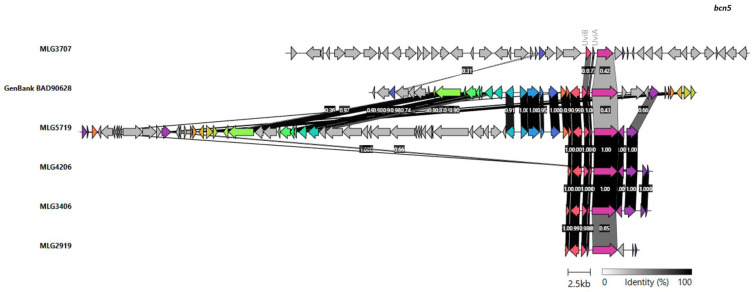
Genetic environments of the *bcn5* gene present in the five *C. perfringens* isolates from our collection and the environment of *bcn5* from the plasmid of reference (accession number BAD90628) from GenBank database. Different genes are indicated with arrows. Colors in the arrows represent the genes, which show similarities, and identities between them are indicated with numbers and a scale of gray. The *bcn5* gene from different bacteria is represented with the purple arrow. The *uviA* and *uviB* genes were located next to the *bcn5* in all the isolates.

The genetic environment of the gene encoding the bacteriocin BC5, *bcn5*, was compared among our isolates and with the reference strain, as presented in Figure 5.

### 3.3. Bacteriocinetic Activity of the Strains

Both methods used to detect antimicrobial activity by the *C. perfringens* strains (spot-on-the-lawn and agar well diffusion methods) gave negative results with the conditions and indicator bacteria used. No antimicrobial activity was detected in any of the *C. perfringens* isolates.

## 4. Discussion

Resistance to antibiotics happens frequently among *C. perfringens* isolates. Tetracycline resistance is very common, both among our isolates and in other studies [20,22]. Ampicillin resistance is not as frequent but has been previously reported [51].

Different tetracycline resistance genes were found in 16 of our isolates. The presence of *tet* genes has been reported in *C. perfringens* isolates in other studies using WGS and appear to be commonly involved in isolates implicated in all types of infections [3]. The *InuP* gene, which encodes a lincosamide nucleotidyltransferase associated with lincomycin resistance [51] was found in three isolates from our collection, two of them resistant to clindamycin.

Macrolide resistance has been previously detected in *C. perfringens*, carrying the gene *erm*(B) [20]. There are three pathways for the acquisition of macrolide resistance: target site modification, efflux pump, and drug inactivation. Target site modification is mediated by 23S rRNA methylation enzymes encoded by *erm* genes, conferring resistance to macrolides [52,53]. Interestingly, in our study, the *erm*(T) gene was detected in two of our strains (*C. perfringens* MLG1108 and MLG 7009), being the first report to find *erm*(T) in *C. perfringens* isolates. Both strains were phenotypically resistant to erythromycin (MIC > 128 μg/mL). Nevertheless, another additional *C. perfringens* isolate (MLG3111) showed resistance to this antimicrobial agent (MIC > 128 μg/mL), although no genes associated with erythromycin resistance were detected by WGS. The mechanisms of macrolide resistance in this isolate should be analyzed in the future to see if it could carry a new mechanism of resistance.

The gene *erm*(T) has been previously detected in other Gram-positive bacteria, such as *Enterococcus spp.*, *Streptococcus spp.*, or *Staphylococcus spp.* [54,55,56,57] but never before in *C. perfringens*. Genetic environments of the *erm*(T) gene have been described in other species [54]. Overall, we can see that the *erm*(T) gene is highly preserved among different species, but its genetic environments are very different from one species to another.

Anaerobic bacteria such as *C. perfringens* usually present low susceptibility to aminoglycosides as they present intracellular reduced transport of the antibiotic [3]. *C. perfringens* MLG2314 harbors the *ant(6)-Ib* gene, which is associated with streptomycin resistance. This gene has previously been reported in toxinotype C [3]. This is the first report in which *ant(6)-Ib* gene is reported in a toxinotype A *C. perfringens* isolate. 

Toxinotyping revealed toxinotypes A and G among our isolates, carrying different toxins. In addition to toxin production, *C. perfringens* is known to produce a variety of other virulence factors. The *cloSI* and *colA* genes, present in most of our isolates, except in *C. perfringens* MLG7307, encode alpha-clostripain and kappa-toxin respectively. Alpha-clostripain is a cysteine endopeptidase. It has been shown to not be essential for disease development [58,59] Kappa-toxin is a clostridial collagenase that actively degrades host tissues to support growth, survival, and dissemination in infected hosts, or to potentiate other toxins by facilitating their diffusion [60]. The gene *colA* is considered a housekeeping gene for MLST. It is interesting to note that *C. perfringens* MLG7307 did not carry it, which, together with other characteristics of the isolate, made it unique in the collection.

Other virulence factors were frequently present, e.g., mu-toxin encoded by the genes *nagH*, *nagI*, *nagJ*, *nagK*, and *nagL*, and sialidases, encoded by *nanH*, *nanI*, and *nanJ*. Mu-toxin consists of hyaluronidases that facilitate the degradation of polysaccharides, such as hyaluronic acid and chondroitin sulfate, thus helping the microorganism to spread into deeper tissues [60,61]. In our isolates, we frequently detect *nagH*, *nagI*, *nagJ*, *nagK*, and, with lesser frequency, *nagL*. The three sialidases NanH, NanI, and NanJ were also highly abundant in our isolates. They are considered to be important virulence factors that promote the pathogenesis of *C. perfringens*; among them, NanI promotes colonization in the intestinal tract and enhances cytotoxic activity [62].

Among the isolates from our study, we detected pDel1, pDel2, pDel3, and pDel4 plasmids, previously described in *C. perfringens* Del1, a strain from a chicken affected by NE, in which the *netB* gene was included within pDel1 [63]. However, in our study, we found pDel1 in three of our type G isolates, but not in all isolates that carried *netB* gene, belonging to type G toxinotypes. Instead, all *C. perfringens* type G isolates carrying *netB* harbored the pDel4 plasmid, suggesting that, in our isolates, *netB* may reside in this plasmid. Our isolates also harbored pLLY_N11_3, a plasmid previously detected by [63], pCP15_3, which has been frequently detected in *C. perfringens* in other studies, and pCPCPI53k-r1_2, which has not yet been studied [64].

Among poultry, the most common STs previously found were ST143 and ST215 [23]. Among our isolates, ST21, ST73, and ST279 were detected. The other three isolates presented two new allelic combinations (*C. perfringens* MLG1619 and MLG1819 with the same allelic combination, and MLG0418 with a different one). In addition, six isolates presented four new STs, showing new housekeeping alleles. These results reveal a high level of diversity among the *C. perfringens* isolates in our collection. In contrast, studies on MLST of *C. perfringens* from poultry indicate that *C. perfringens* isolates from NE diseased birds and healthy birds within outbreaks tend to be closely related. Even though *C. perfringens* is very diverse, there are subpopulations of *C. perfringens* types commonly found in NE birds that are not as variable as those found in healthy chickens [64]. Among the STs of our isolates, only ST21 has previously been reported in NE chickens [65]. In addition, we found new allelic combinations and STs, highlighting that most of our strains are from undescribed STs, thus adding more variability to *C. perfringens* from chickens affected by NE.

All isolates carried genes of sactipeptides (sulfur-alpha carbon thioether crosslinked peptides), a novel type of lantibiotic that presents various biological activities such as antibacterial, spermicidal, and hemolytic properties. However, their function is still being studied [66]. Genes encoding lassopeptides were also found in three of the isolates. They belong to a specific family of RiPPs with an unusual lasso structure. Lasso peptides possess remarkable thermal and proteolytic stability and diverse biological activities such as antimicrobial activity, enzyme inhibition, receptor blocking, anticancer properties, and HIV antagonism. They have promising potential therapeutic effects in gastrointestinal diseases, tuberculosis, Alzheimer’s disease, cardiovascular diseases, fungal infections, and cancer [67,68].

The gene encoding bacteriocin BCN5 was detected in five of our *C. perfringens* isolates. It is a plasmid-encoded bacteriocin with promising activity against *Mycobacterium tuberculosis* [69]. Its activation has been shown to depend in vivo and in vitro on the activity of the UviA and UviB proteins [70], and it is inducible by UV irradiation [71]. The phylogenetic relationships of bacteriocin BNC5 of our stains and those included in Genbank revealed that BNC5 of three of our *C. perfringens* isolates (MLG3406, MLG4206, and MLG5719) were identical among themselves, as well as to bacteriocin BCN5 P08696 from the GenBank database, which corresponds to the bacteriocin first described and characterized [71]. Moreover, the BCN5 sequence of the remaining two *C. perfringens* isolates of our collection and the bacteriocin BNC5 BAD90628 from the GenBank database [71] clustered separately (Figure 5). With regard to the alignments of bacteriocin BCN5, the low level of identity between them may be evidence that they are different bacteriocins. Overall, bacteriocin BCN5 was identical to and well conserved within three of the *C. perfringens* isolates from our collection and the bacteriocin BCN5 P08696 of reference, whereas it presented polymorphisms with the other isolates of our collection and with the other BCN5 of reference (BAD90628). The genetic environments of the contigs containing the *bcn5* gene of our isolates and the reference plasmid BAD90628 showed many differences among them, whereas the genetic environments of *C. perfringens* MLG2919, MLG3406, and MLG4206 were similar. *C. perfringens MLG5719* showed similarities with the plasmid of reference BAD90628, with two insertions. The *C. perfringens* MLG7307 showed no similarity to the others. As no plasmids were identified in strain MLG7307, the *bcn5* gene in this strain could be located at the chromosome.

Surprisingly, although many genes encoding for antimicrobial peptide production have been detected, no antimicrobial activity was observed in *C. perfringens* isolates against the indicator strains used. This is probably because the presence of encoding genes detected by WGS does not necessary lead to gene expression and to synthesis of the antimicrobial protein, perhaps requiring specific conditions for gene expression. This phenomenon has already been described for others bacteriocins, such as nisin and microcins [72,73]. Future studies should investigate this issue.

## 5. Conclusions

*C. perfringens* infection of avian species is a serious animal health concern. Among all the characteristics studied in the 20 isolates from NE poultry of toxinotypes A and G, we could highlight the presence of the *erm*(T) gene, reported only in other Gram-positive bacteria, including ARG, as well as the presence of multiple toxins and virulence factors, and the existence of variability among the ST of the isolates. In addition, of great interest is the detection of genes encoding different bacteriocins, with BCN5 being of relevance. 

Moreover, it should also be noted that the *C. perfringens* MLG7307 isolate was clearly distinct from the other strains. This strain was phenotypically resistant to clindamycin and cefotaxime, and it had intermediate susceptibility to ampicillin, although no resistance genes were observed by WGS. It also had a combination of toxins that made it impossible to toxinotype. Interestingly, even though it was identified as *C. perfringens*, it lacked the housekeeping gene *colA*. Further studies should be carried out on this strain to determine its characteristics and possible classification.

Concluding, WGS analysis provides insights into the genomic characteristics of bacteria and is a promising tool for the study and better understanding of the avian pathogen *C. perfringens*.

## Figures and Tables

**Table 1:** MICs values (in μg/mL) for the collection of 20 *C. perfringens* isolates.

*C. perfringens* Isolate	Resistance Phenotype	TET	CLI	AMP	CTX	CHL	MTZ	IPM	ERY
MLG0418	Susceptible	2	<0.25	<0.25	2	4	4	<0.25	16
MLG2203	Susceptible	<0.25	<0.25	<0.25	<0.25	4	2	<0.25	8
MLG4201	TET	16	2	<0.25	1	4	2	<0.25	16
MLG5719	TET	8	2	<0.25	2	4	4	<0.25	8
MLG5806	TET	16	2	<0.25	<0.25	4	8	<0.25	16
MLG7814	TET	32	2	<0.25	1	4	2	<0.25	8
MLG1819	CLI	<0.25	4	<0.25	1	4	8	<0.25	16
MLG1619	CLI	<0.25	8	<0.25	4	4	8	<0.25	16
MLG6907	CLI	4	8	<0.25	<0.25	4	4	<0.25	8
MLG4206	CLI	4	4	<0.25	1	4	4	<0.25	1
MLG0618	TET, CLI	32	4	<0.25	2	4	2	<0.25	8
MLG0712	TET, CLI	16	8	<0.25	2	4	4	<0.25	16
MLG2314	TET, CLI	16	>128	<0.25	4	8	4	1	16
MLG2919	TET, CLI	64	>128	<0.25	2	8	8	0.5	8
MLG3406	TET, CLI	8	4	<0.25	1	4	4	<0.25	16
MLG7309	TET, CLI	16	4	<0.25	1	4	1	<0.25	4
MLG3111	TET, ERY	16	1	<0.25	0.5	4	1	<0.25	>128
MLG1108	TET, CLI, ERY	8	>128	<0.25	1	4	4	<0.25	>128
MLG7009	TET, CLI, ERY	16	>128	0.5	2	4	8	<0.25	>128
MLG7307	CLI, AMP, CTX	2	32	1	64	4	8	2	4

TET: tetracycline; CLI: clindamycin; AMP: ampicillin; CTX: cefotaxime; CHL: chloramphenicol; MTZ: metronidazole; IMP: imipenem; ERY: erythromycin. Red cells indicate resistance values (R); yellow cells indicate intermediate values (I) according to the CLSI standards. White cells indicate the susceptible category. Note: For ERY, there are no breakpoints to establish susceptibility by CLSI. We consider as resistant the isolates with an MIC higher than 128 μg/mL.

**Table 2:** Resistance phenotype and genotype of the *C. perfringens* collection.

*C. perfringens* Isolate	Resistance Phenotype ^a^	Resistance Genotype Detected		
		Resistance Genes	Identity	Accession Number ^c^
MLG0418	Susceptible Susceptible	*tetA*	99.17	L20800
MLG2203		No genes detected		
MLG4201	TET	*tetA*	99.84	AB001076
		*tetB*	99.74	NC_010937
		*InuP*	99.8	FJ589781
MLG5719	TET	*tetA*	100	AB001076
		*tetB*	99.74	NC_010937
MLG5806	TET	*tetA*	100	AB001076
		*tetB*	99.74	NC_010937
MLG7814	TET	*tetA*	99.84	AB001076
		*tetB*	99.74	NC_010937
		*lnuP*	99.8	FJ589781
MLG1819	CLI	No genes detected		
MLG1619	CLI	No genes detected		
MLG6907	CLI	*tetA*	99.26	AB001076
MLG4206	TET, CLI	*tetA*	100	AB001076
		*tetB*	99.74	NC_010937
MLG0618	TET, CLI	*tetA*	99.84	AB001076
		*tetB*	99.74	NC_010937
		*lnuP*	99.8	FJ589781
MLG0712	TET, CLI	*tetA*	99.84	AB001076
		*tetB*	99.74	NC_010937
MLG2314	TET, CLI	*tetA*	99.18	AB001076
		*tet(44)*	98.75	NZ_ABDU01000081
		*lnuP*	99.8	FJ589781
		*ant(6)-Ib*	100	FN594949
MLG2919	TET, CLI	*tetA*	100	AB001076
		*tetB*	99.67	NC_010937
MLG3406	TET, CLI	*tetA*	100	AB001076
		*tetB*	99.74	NC_010937
MLG7309	TET, CLI	*tetA*	99.84	AB001076
		*tetB*	99.74	NC_010937
MLG3111	TET, ERY	*tetA*	99.84	AB001076
		*tetB*	99.74	NC_010937
		*lnuP*	99.8	FJ589781
MLG1108	TET, CLI, ERY	*tetA*	99.84	AB001076
		*tetB*	99.74	NC_010937
		*erm*(T)	99.86	AY894138
MLG7009	TET, CLI, ERY	*tetA*	99.84	AB001076
		*tetB*	99.74	NC_010937
		*erm*(T)	99.86	AY894138
MLG7307	CLI, AMP ^b^, CTX	No genes detected		

^a^ TET: tetracycline; CLI: clindamycin; AMP: ampicillin; CTX: cefotaxime; ERY: erythromycin; ^b^ intermediate susceptibility; ^c^ accession number of the resistance gene used in the comparison.

**Table 3:** Sequence type and new allelic combinations of the 20 *C. perfringens* isolates.

Sequence Types (ST)									
Strain	ST	Housekeeping Genes							
		*colA*	*groEL*	*gyrB*	*nadA*	*pgk*	*plc*	*sigk*	*sodA*
*C. perfringens* MLG0712	21	3	1	3	1	1	4	2	3
*C. perfringens* MLG1108	21	3	1	3	1	1	4	2	3
*C. perfringens* MLG7009	21	3	1	3	1	1	4	2	3
*C. perfringens* MLG7309	21	3	1	3	1	1	4	2	3
*C. perfringens* MLG0618	73	39	19	3	1	1	4	5	1
*C. perfringens* MLG3111	73	39	19	3	1	1	4	5	1
*C. perfringens* MLG4201	73	39	19	3	1	1	4	5	1
*C. perfringens* MLG7814	73	39	19	3	1	1	4	5	1
*C. perfringens* MLG2314	279	77	41	8	1	4	1	19	1
**New or Unknown ST ^a^**									
Strain	ST ^b^ (Nearest ST)	Housekeeping Genes							
		*colA*	*groEL*	*gyrB*	*nadA*	*pgk*	*plc*	*sigk*	*sodA*
*C. perfringens* MLG0418	NAC (53)	37	22	17	28	1	27	18	19
*C. perfringens* MLG1619	NAC (629)	6	1	3	13	1	4	2	3
*C. perfringens* MLG1819	NAC (629)	6	1	3	13	1	4	2	3
*C. perfringens* MLG2203	New (131)	41	44	37 ^c^	47 ^c^	18	101 ^c^	25 ^c^	38 ^c^
*C. perfringens* MLG 2919	New (625)	6 ^c^	5	24	1	7	33	4	1
*C. perfringens* MLG3406	New (340, 613)	3	6	1	1	4	43	5	71 ^c^
*C. perfringens* MLG4206	New (340, 613)	3	6	1	1	4	43	5	71 ^c^
*C. perfringens* MLG5719	New (340, 613)	3	6	1	1	4	43	5	71 ^c^
*C. perfringens* MLG5806	New	3 ^c^	56 ^c^	29 ^c^	49	8	88 ^c^	5	79 ^c^
*C. perfringens* MLG6907	unknown (200)	4	1	3	13	1	109	80 ^d^	20
*C. perfringens* MLG7307	Unknown	No hit ^e^	121	83	135	63	163	87	125

^a^ In this section are recorded (a) new allelic combinations (NACs), (b) potential new STs, because some of the gene sequences showed differences with those registered in MLST database (the closest allele is included), and (c) unknown STs, because incomplete coverage or lack of some alleles occurred. ^b^ The type of ST is recorded as NAC (new allelic combination), new ST (new sequence for any of the intrinsic genes), or unknown ST (not complete sequence of any of the intrinsic genes or lack of any of the genes). The closest ST/STs are also included, when possible. ^c^ Alleles with <100% identity. ^d^ Alleles with <100% coverage. ^e^ No hit: this strain lacked the housekeeping gene and ST could not be defined.

**Table 4:** Secondary metabolites detected in the *C. perfringens* isolates.

*C. perfringens* Isolate	Secondary Metabolites
MLG0418	Sactipeptides
MLG0618	Sactipeptides
MLG0712	Sactipeptides
MLG1108	Sactipeptides
MLG1619	Sactipeptides
MLG1819	Sactipeptides
MLG2203	Sactipeptides
MLG2314	Sactipeptides
MLG2919	Sactipeptides, lasso-peptides, bacteriocin BCN5
MLG3111	Sactipeptides, RiPP-like
MLG3406	Sactipeptides, RiPP-like, bacteriocin BCN5
MLG4201	Sactipeptides, NRPS-like
MLG4206	Sactipeptides, bacteriocin-like, bacteriocin BCN5
MLG5719	Sactipeptides, bacteriocin BCN5, NRPS-like
MLG5806	Sactipeptides, NRPS-like
MLG 6907	Sactipeptides, lasso-peptides
MLG7009	Sactipeptides
MLG7307	Sactipeptides, lasso-peptides, bacteriocin BCN5
MLG 7309	Sactipeptides
MLG7814	Sactipeptides

## Data Availability

Not applicable.

## Supplementary Materials

The following supporting information can be downloaded at: https://www.mdpi.com/article/10.3390/pathogens12070905/s1, Figure S1: Genetic alignments of the *erm*(T) methylases of *C. perfringens* MLG1108 and MLG7009 with *erm*(T) methylases from other species; Figure S2: Alignment of the bacteriocin BCN5 from our isolates (*C. perfringens* MLG3406, MLG4206, MLG5719, MLG2919 and MLG7307) and two bacteriocin BCN5 from GenBank database (P08696 and BAD90628).
