# Supplementary material for: Pathogenicity and Antibiotic Resistance Diversity in Clostridium perfringens Isolates from Poultry Affected by Necrotic Enteritis in Canada
Source: Pathogens. 2023 Jul 3;12(7):905. doi: 10.3390/pathogens12070905 (PMC10383762; doi:10.3390/pathogens12070905)

Supplementary Figure S1. Genetic alignments of the *erm*(T) methylases of *C. perfringens* MLG1108 and MLG7009 with *erm(T)* methylases from other species

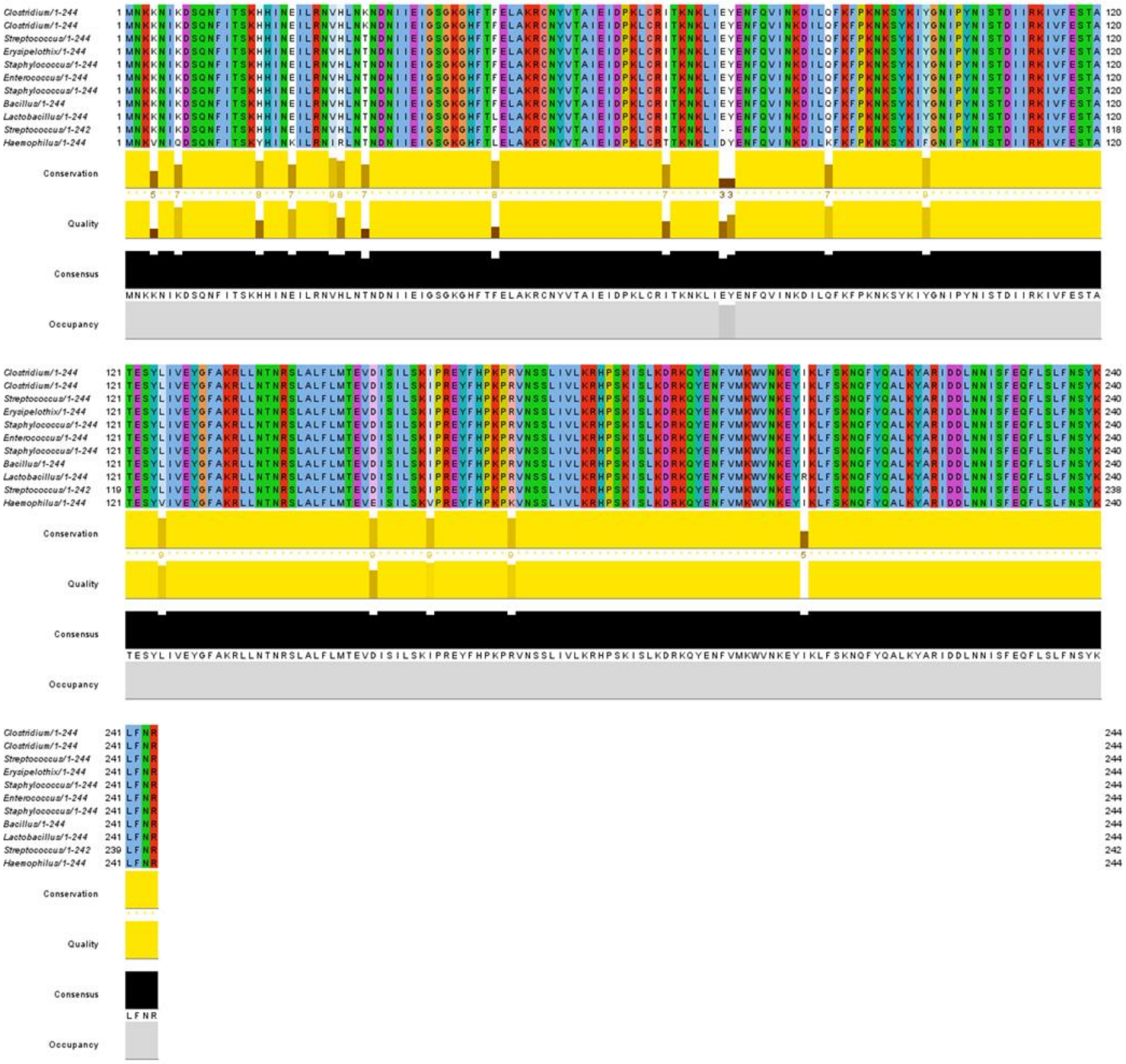

Supplementary Figure S2. Alignment of the bacteriocin BCN5 from our isolates (*C. perfringens* MLG3406, MLG4206, MLG5719, MLG2919 and MLG7307) and two bacteriocin BCN5 from GenBank database (P08696 and BAD90628)

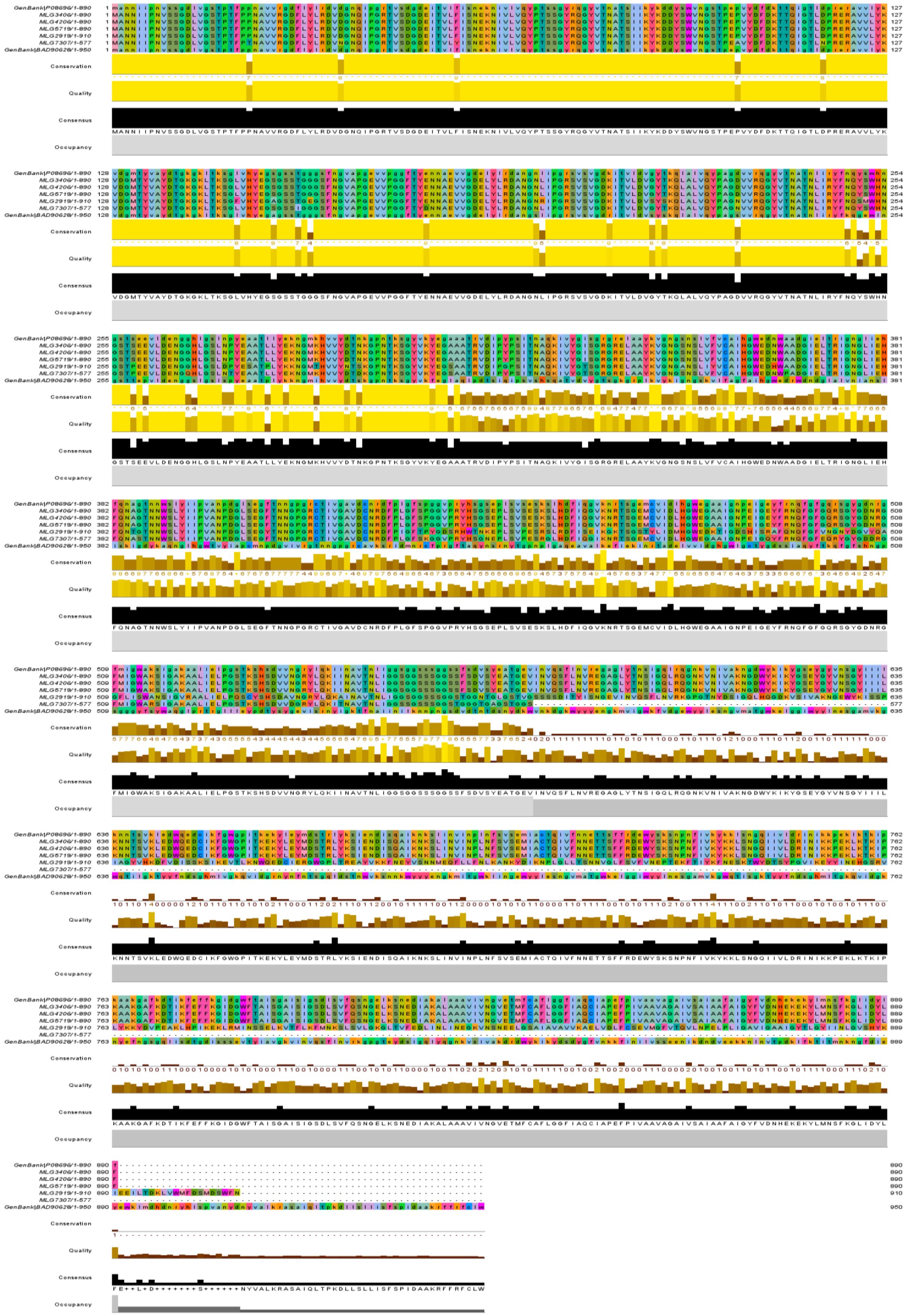

Supplement: Supplementary file 1 [file pathogens-12-00905-s001.zip › pathogens-2417107-supplementary.pdf]
